# General and domain-specific cognitive reserve, mild cognitive impairment, and dementia risk in older women

**DOI:** 10.1016/j.trci.2019.02.003

**Published:** 2019-04-10

**Authors:** Andrew J. Petkus, Susan M. Resnick, Stephen R. Rapp, Mark A. Espeland, Margaret Gatz, Keith F. Widaman, Xinhui Wang, Diana Younan, Ramon Casanova, Helena Chui, Ryan T. Barnard, Sarah Gaussoin, Joseph S. Goveas, Kathleen M. Hayden, Victor W. Henderson, Bonnie C. Sachs, Santiago Saldana, Aladdin H. Shadyab, Sally A. Shumaker, Jiu-Chiuan Chen

**Affiliations:** a Department of Neurology, University of Southern California, Los Angeles, CA, USA; b National Institute on Aging, Laboratory of Behavioral Neuroscience, Baltimore, MD, USA; c Department of Psychiatry and Behavioral Medicine, Wake Forest School of Medicine, Winston-Salem, NC, USA; d Department of Social Sciences and Health Policy, Wake Forest School of Medicine, Medical Center Blvd, Winston-Salem, NC, USA; e Department of Biostatistical Sciences, Wake Forest School of Medicine, Medical Center Blvd, Winston-Salem, NC, USA; f Center for Economic and Social Research, University of Southern California, Los Angeles, CA, USA; g Graduate School of Education, University of California, Riverside, CA, USA; h Department of Preventive Medicine, University of Southern California, Los Angeles, CA, USA; i Department of Psychiatry, Medical College of Wisconsin, Tosa Health Center, Milwaukee, WI, USA; j Department of Health Research & Policy (Epidemiology), Stanford University, Stanford, CA, USA; k Department of Neurology and Neurological Sciences, Stanford University, 259 Campus Drive, Stanford, CA, USA; l Department of Neurology, Wake Forest School of Medicine, Medical Center Blvd, Winston-Salem, NC, USA; m Department of Family Medicine and Public Health, University of California, San Diego School of Medicine, La Jolla, CA, USA

**Keywords:** Cognitive reserve, Dementia, Mild cognitive impairment, Structural equation modeling

## Abstract

**Introduction:**

In a geographically diverse sample of women, we asked whether cognitive reserve (CR) is best viewed as a general or cognitive domain-specific construct and whether some cognitive reserve domains but not others exert protective effects on risk of developing mild cognitive impairment (MCI) or dementia.

**Methods:**

Estimates of general and domain-specific CR were derived via variance decomposition in 972 cognitively intact women from the Women's Health Initiative Study of Cognitive Aging and Women's Health Memory Study Magnetic Resonance Imaging. Women were then followed up for 13 years.

**Results:**

General CR was the strongest predictor of reduced risk for both MCI and dementia, compared to domain-specific CR measures. Verbal memory, figural memory, and spatial CR were independently protective of MCI, but only verbal memory was independently associated with reduced risk for dementia.

**Discussion:**

Cognitive reserve is a heterogenous construct with valid quantitative measures identifiable across different neuropsychological processes associated with MCI and dementia.

## 1 Background

Despite the well-established association between neuropathological processes and cognitive aging [1], discrepancies between detected neuropathology and clinical manifestation are common [2]. Cognitive reserve (CR), a neuropsychological construct representing a dynamic process influenced by an individual's combined life experience, purportedly explains some of these observed discrepancies [3]. Quantifying CR is challenging and historically lacked precision [4]. Researchers have typically estimated CR using imprecise sociobehavioral proxy measures [3] (e.g., educational and occupational attainment, vocabulary tests), that are static, and qualitatively vary in populations from different geographical regions [5] and across cohorts.

The residual approach is a recently developed method to quantify CR [6] that offers potential advantages [3] over sociobehavioral proxy measures. Under this approach, CR represents the residual variance in cognitive performance after regressing out the effects of neuropathological factors, sociodemographic characteristics, and measurement errors [6]. Prior studies using this statistical approach have largely used structural magnetic resonance imaging (MRI) data, including estimates of global brain volume, hippocampal volume, and indices of small vessel ischemic disease [6], [7], [8], [9], as all of these structural MRI variables have been associated with cognitive decline [10], [11], [12]. Earlier single-site studies showed that verbal memory CR was associated with less conversion from mild cognitive impairment (MCI) to dementia [6], [7], [8].

Because measures of cognitive performance in multiple domains are highly correlated with each other, the existence of a general factor (“g”) underlying the cognitive reserve construct likely exists [13]. Few studies had attempted to derive the general factor underlying CR using the variance decomposition method [9], [14], and no prior studies have examined the association between general CR (defined in a cognitively intact population) and subsequent risk of MCI or dementia. Conversely, among individuals who are cognitively intact, there is significant differentiation between cognitive abilities, as general ability explains less of the variance in performance within each domain [15]. This differentiation of cognitive abilities highlights the importance of examining CR specific to a number of domains (e.g., attention, figural memory, language, visuospatial) instead of solely general CR or episodic memory CR [16], [17] in cognitively intact individuals. It remains unclear, however, to what extent multidomain CR measures may be identifiable with differential protection against MCI or dementia, especially in large geographically diverse samples.

To address these critical gaps in knowledge, we examined the structure of general and domain-specific CR estimates derived via variance decomposition and examined the resulting CR estimates in relation to prospective MCI and dementia in a geographically diverse cohort of well-characterized, community-dwelling cohort of older women. A number of sex differences exist in the expression of dementia, including a higher prevalence for women [18]; thus, understanding the association of CR with cognitive outcomes in women is particularly important. The first aim was to estimate general and cognitive domain–specific reserve and to formally test their factor structure. The second aim was to demonstrate the construct validity of the resulting CR estimates by examining their respective associations with risk of developing MCI and dementia among older women who were cognitively intact at the time of measuring CR.

## 2 Methods

### 2.1 Participants

**Fig. 1:**
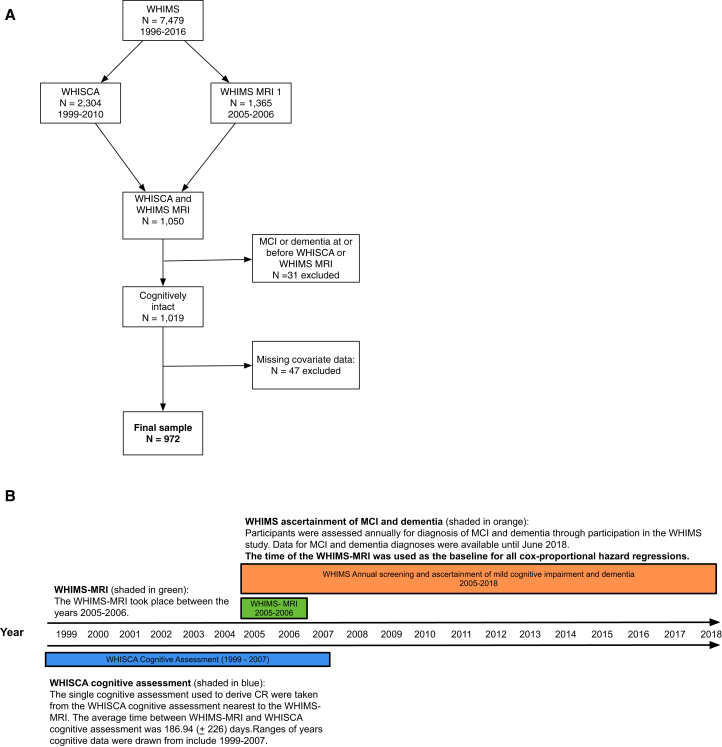
Diagram of study participation and timeline of when assessments were administered. Fig. 1A is a flowchart of study participation with exclusion criteria. Fig. 1B provides a timeline of when assessments were administered. Abbreviations: CR, cognitive reserve; MCI, mild cognitive impairment; MRI, magnetic resonance imaging; WHISCA, Women's Health Initiative Study of Cognitive Aging; WHIMS, Women's Health Initiative Memory Study.

Data for this study included 972 community-dwelling women from the Women's Health Initiative Memory Study (WHIMS) [19] who also participated in both the Women's Health Initiative Study of Cognitive Aging (WHISCA) [20] and the WHIMS Magnetic Resonance Imaging (WHIMS-MRI) [21] Study (Fig. 1A) ancillary studies. The WHIMS study (N = 7479) began in 1996 and was an ancillary study to the Women's Health Initiative (WHI) clinical trial of Hormone Therapy [22]. The WHIMS cohort continues to be followed up annually. WHISCA was an ancillary study to WHIMS in which a subsample of 2304 cognitively intact women annually completed a thorough neurocognitive battery between the years of 1999 and 2010. During the years of 2005 and 2006, a subset of 1365 women from WHIMS underwent brain MRI imaging through participation in the WHIMS-MRI study. A total of 1050 women participated in both WHIMS-MRI and WHISCA. All women provided informed consent for participation. For this study, we used neurocognitive data from the WHISCA assessment that was closest to the date of the WHIMS-MRI. Fig. 1B presents a timeline of when the cognitive assessment and WHIMS-MRI were completed. We excluded women who were already classified as MCI or dementia at the time of either the WHISCA or MRI assessment (n = 31) and those with missing data on covariates of interest (n = 47), resulting in a final sample of 972. Supplemental Table 1 provides a comparison of WHISCA participants who were included in these analyses and those who were excluded. Women who participated in both WHISCA and WHIMS-MRI were generally younger, physically and cognitively healthier than women who did not participate in both studies.

### 2.2 Assessment of cognitive performance

The WHISCA neurocognitive battery measured the following cognitive domains: attention, verbal memory, figural memory, language, and spatial ability (Supplementary Methods). All indices were transformed and standardized as z-scores based on the initial WHISCA mean and SD, with higher scores representing better performance. Composite scores for each domain were created by computing the average score across all tests within a domain.

### 2.3 Structural MRI assessment

MRI scanning was conducted using standardized protocols [23], [24], [25]. The primary measures of brain volume (in cm^3^) used in the present study were total normal brain volume (total; grey matter), hippocampal volumes (left; right), total volume of small vessel ischemic diseases (SVIDs), and intracranial volume. These brain MRI variables were selected as they are representative of global brain integrity, are associated with cognitive performance, and to be consistent with prior studies [6], [7], [8]. Additional information about imaging procedures is provided in the Supplementary Methods.

### 2.4 Classification of mild cognitive impairment and dementia

The present study included two clinical end points of cognitive impairment: MCI based on Petersen's criteria [26] and all-cause dementia defined by the Diagnostic and Statistical Manual of Mental Disorders, Fourth Edition (DSM-4) [27] criteria. For annual screenings conducted in 39 WHIMS sites (including satellite clinics), centrally trained/certified and masked interviewers administered the Modified Mini Mental Status (3MS) examination [28] during each clinic visit. Women who screened positive using age-/education-adjusted 3MS scores were administered extensive neuropsychological testing (including the Consortium to Establish a Registry for AD battery [29]) and behavioral symptoms/function assessment [30]. Beginning in 2008, annual cognitive screenings were conducted by telephone [31] and the data from this battery were used to adjudicate MCI and dementia status. The cognitive measures used to compute CR were not used in making the MCI or dementia diagnosis. A committee of experts blinded to participant's cognitive reserve status assigned the diagnostic classification. Data on MCI and dementia status were available up to June 2018.

### 2.5 Assessment of covariates

A structured questionnaire was administered at the baseline visit to gather information on demographics (age, race/ethnicity), socioeconomic factors (education, family income, employment status), and lifestyle factors (smoking and alcohol use, physical activity). Depressive symptoms were measured with the 15-item Geriatric Depression Scale [32]. Clinical characteristics were ascertained, including postmenopausal hormone treatment, history of cardiovascular disease (including previous coronary heart, stroke, or transient ischemic attack), hypertension (defined as elevated blood pressure [systolic ≥ 140 or diastolic ≥ 90] or use of antihypertensive medication), calculated body mass index, and diabetes mellitus (defined as physician diagnosis plus oral medications, or insulin therapy). Good reliability and validity of the self-reported medical histories and the physical measures have been previously documented [33].

### 2.6 Variance decomposition approach to quantify reserve

Structural equation models (SEMs) were constructed to decompose the variance in performance within cognitive domain contributed by MRI-inferred neuropathological factors, sociodemographic characteristics, and measurement error, with the resulting residual hypothesized to represent cognitive reserve. The theoretical model first proposed by Reed et al. [6] was used as a guide for these analyses with minor deviations. A more detailed description of the SEMs with rationale for deviations from the Reed approach [6] is provided in the Supplementary Methods. Specifically, the composite score from each cognitive domain was regressed on age, latent composite estimates of hippocampal volume, global brain volume, SVID, and sociodemographic factors (variance explained by education and race/ethnicity) while accounting for 15% of the variance as measurement error. A higher score on the reserve factor would indicate that cognitive performance in the respective domain was greater than what would have been expected, given the individual's profiles of predictors including age, sociodemographic characteristics, and brain MRI variables. The mean of the reserve factor was z-score standardized (mean = 0; SD = 1). The SEMs also specified that the reserve factor was orthogonal to the neuropathology and demographic factors.

**Fig. 2:**
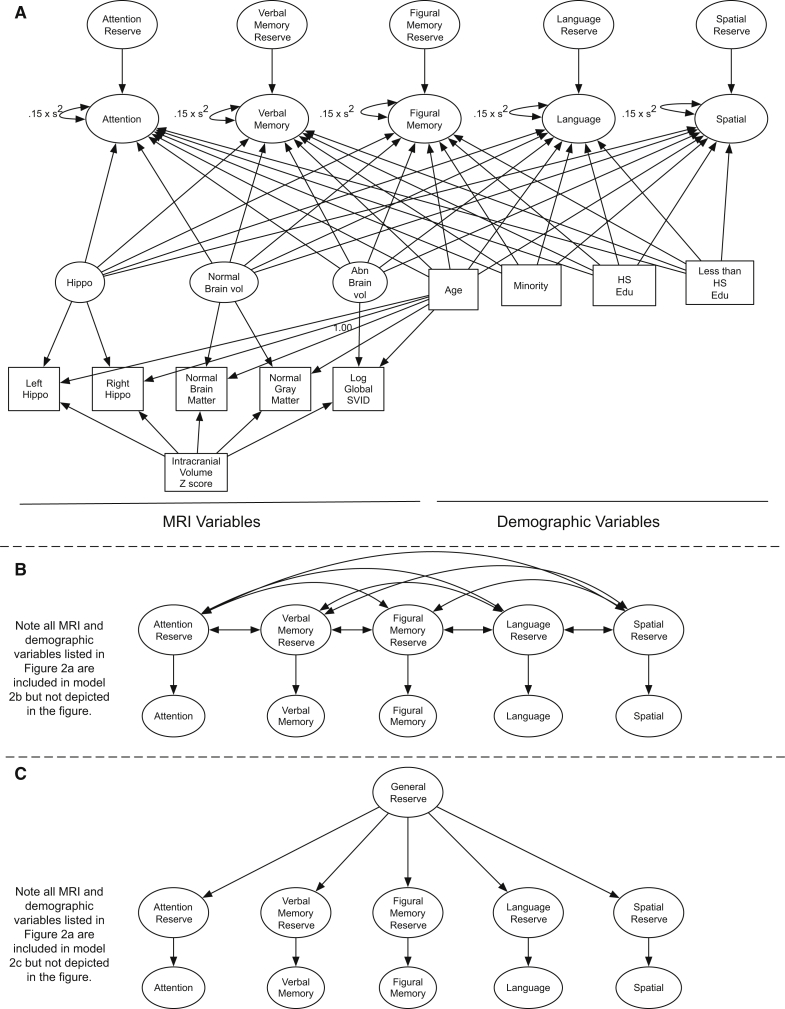
Diagram of the three multivariate variance decomposition models that were fit to quantify reserve across cognitive domains. Fig. 2A depicts the multivariate first-order orthogonal factor model. Fig. 2B depicts the first-order multivariate correlated reserve model. Note this model also includes the same structural MRI and demographic indicators as presented in Fig. 2A. Fig. 2C depicts the higher-order multivariate reserve model. This model also includes the same structural MRI and demographic indicators as presented in Fig. 2A. These components were omitted to simplify the presentation. Fig. 2 notes: Left Hippo represents left hippocampal volume, Right Hippo represents right hippocampal volume, Log SVID represents log-transformed small vessel ischemic disease. All MRI variables had residual variance constrained to 0.15 times the variance to account for error in measurement. Abbreviations: MRI, magnetic resonance imaging; SVID, small vessel ischemic disease.

We started with a univariate SEM where each domain-specific reserve was modeled separately, to ensure that each domain could be estimated with adequate model fit before moving to multivariate modeling. The multivariate SEMs then proceeded in three incremental steps: (1) a first-order multivariate orthogonal factor SEM (Fig. 2A) including all domain-specific factors estimated simultaneously in the same model with no correlation among the reserve factors; (2) a first-order multivariate SEM with correlated factors (Fig. 2B) including all domain-specific factors estimated simultaneously; and (3) a higher-order multivariate SEM (Fig. 2C), including all domain-specific factors plus a “general cognitive reserve” factor which was a higher-order latent variable that captures variance common across all domains. Comparisons of the fit of the three multivariate models allow a formal test to identify the model that best described the associations among domain-specific reserve estimates. Multiple indices were used to evaluate model fit: the comparative fit index (CFI), the Tucker Lewis Index (TLI), the root-mean-squared error of approximation (RMSEA) with 90% confidence interval (CI), and the chi-square statistics by degrees of freedom. Standard criteria of CFI > 0.95, TLI > 0.95, and RMSEA < 0.06 were used to represent good model fit [34]. All SEM's were fit with the program MPLUS version 8 [35] and the MPLUS automation program [36] in R [37].

### 2.7 Cox-proportional hazard regressions

The resulting estimates of cognitive reserve from each SEM were extracted and stored as indicators that were entered in the Cox proportional-hazards models examining the association between reserve estimates and risks for MCI and dementia. Survival time was calculated from the date of completion of the WHIM-MRI to date of MCI or dementia diagnosis, date of last assessment, death, or June 2018. We first conducted separate Cox models, in which the association between each reserve factor and cognitive impairment was estimated. To determine the relative contribution of each reserve estimate, we then constructed a joint Cox model including all reserve estimates. We did not include the effect of general cognitive reserve in the joint model because general cognitive reserve is a function of all cognitive domains. We adjusted for the time between the cognitive assessment and the MRI by adding the number of days between these assessments as a covariate in all Cox regressions. All models also adjusted for the potential confounding by a list of covariates including the latent factor scores of the brain MRI variables, demographic characteristics (geographic region of residence, age, race/ethnicity, employment status), lifestyle factors (smoking status, alcohol use, physical activities), depressive symptoms, and physical health (diabetes, high cholesterol, hypertension, hormone use ever, and cardiovascular disease). The proportional-hazards assumption was verified with the scaled Schoenfeld residual test. All Cox proportional-hazards models were run with the survival package in R.

### 2.8 Sensitivity analyses

A number of sensitivity analyses were conducted to test the robustness of our findings. To rule out reverse causation and the possibility that any effects of CR on risk of MCI or dementia were driven by an unmeasured neuropathological process, we first ran sensitivity analyses examining the association between CR variables and MCI and dementia status after excluding all MCI cases classified within 5 years of the MRI. Similarly, we reexamined the association between CR and dementia risk after excluding incident dementia cases identified within 5 years of the MRI. Next, we tested our assumption of setting the measurement error on each domain of cognitive performance to 15% of the variance by systematically varying this estimate. Additional SEM models were constructed by specifying zero, 5%, 10%, and 20% error variance. The various estimates of reserve were extracted from the SEM models and these estimated scores were used in proportional-hazard regressions to examine their associations with incident MCI and dementia.

## 3 Results

### 3.1 Participant characteristics and incidence of MCI and dementia

Women were on average 78.1 (SD = 3.83) years old at the time of cognitive assessment, and mostly Caucasian with more than a high school education (see Table 1 for descriptive statistics). The WHISCA cognitive assessments were completed approximately within 6 months (186.97 ± 226.52 days) of receiving brain MRI scans. A total of 156 women (16.05%) developed MCI, while 104 women (10.70%) developed dementia on average 7.12 (SD = 3.04) and 8.26 (SD = 2.69) years after receiving brain MRI scans, respectively. A total of 94 women died during the follow-up period.

**Table 1:** Sample descriptive statistics (N = 972)

Variable	N or mean	% or SD
Age at cognitive assessment (years)	77.27	3.75
Time between cognitive assessment and MRI (days)	186.97	226.52
U.S. region		
Northeast	168	17.28
South	150	15.43
Midwest	393	40.43
West	261	26.85
Education		
Less than high school	35	3.60
High school	206	21.19
More than high school	731	75.21
Ethnicity		
African-American	39	3.91
Hispanic white	13	1.34
White (not of Hispanic)	876	92.18
Other or missing	25	2.57
Lifestyle		
Smoking status		
Never smoked	572	58.85
Past smoker	362	37.24
Current smoker	38	3.91
Moderate or strenuous activities ≥20 minutes		
No activity	532	54.73
Some activity	57	5.86
2–4 episodes/week	210	21.60
≥4 episodes/week	173	17.80
Physical health		
Hormone treatment ever		
No	511	52.57
Yes	461	47.43
Hypertension ever		
No	630	64.81
Yes	342	35.19
Diabetes treated ever (pills or shots)		
No	944	97.12
Yes	28	2.88
High cholesterol requiring pills ever		
No	803	82.61
Yes	169	17.39
Cardiovascular disease ever		
No	834	85.80
Yes	138	14.20
Cognitive performance		
Attention/working memory		
Digit span forward	7.65	2.15
Digit span backward	6.71	2.02
Verbal episodic memory		
CVLT list A 1-3 total	29.42	6.48
CVLT long delay free recall	9.65	3.02
Figural memory		
Number of errors Benton Visual Retention	6.11	3.48
Language		
Phonemic fluency (average words/trial)	14.37	4.13
Category fluency (average words/trial)	14.31	3.21
Visuospatial		
Card rotations	65.98	29.95
Structural MRI volume (cm^3^)		
Right hippocampus	2.76	.43
Left hippocampus	3.08	0.41
Total brain volume, normal	839.92	76.62
Global small-vessel ischemic disease volumes	7.01	9.82

Abbreviations: CVLT, California Verbal Learning Test; MRI, magnetic resonance imaging.

### 3.2 Reserve SEM models

All five univariate SEM models had good model fit (CFI > 0.99, TLI ≥ 0.98, RMSEA ≤ 0.04; Supplemental Table 2). The multivariate orthogonal first-order factor model had unacceptable model fit based on the TLI and RMSEA (*χ*^2^ (45) = 244.57, CFI = 0.96, TLI = 0.90, RMSEA = 0.07 [90% CI = 0.06 to 0.08]). The multivariate first-order correlated factor SEM model was significantly better fitting than the first-order orthogonal model and with the exception of the TLI met criteria for good model fit (*χ*^*2*^ (35) = 128.47, CFI = 0.98, TLI = 0.94, RMSEA = 0.05 [90% CI = 0.04–0.06]; Δ *χ*^*2*^ = 116.10, Δ df = 10, *P* < .01). Although fitting less well than the first-order correlated factors model (Δ *χ*^*2*^ = 34.95; Δ df = 5, *P* < .01), the higher-order multivariate model exhibited and met criteria for good model fit based on all fit indices except the TLI (*χ*^*2*^ (40) = 163.92, CFI = 0.97, TLI = 0.93 RMSEA = 0.056 [90% CI = 0.05–0.07]). The general cognitive reserve factor accounted for moderate amounts of variance in domain-specific reserve estimates, although higher with figural memory, verbal memory, and language than with attention and visuospatial ability: 20.0% [95% confidence interval = 13.7%–27.6%] for attention; 29.4% [95% CI = 21.8%–38.1%] for verbal memory, 47.1% [95% CI = 37.2%–58.1%] for figural memory, 32.2% [95% CI = 24.0%–41.6%] for language, and 23.4% [95% CI = 16.6%–31.4%] for spatial ability (see Supplementary Table 3 for standardized parameter estimates from the higher-order model). Pearson's correlations among the reserve estimates ranged from r = 0.15 between attention and spatial ability to 0.43 between verbal and figural memory (see Supplementary Table 4 for the full correlation matrix). The observed correlation structure among reserve factors did not vary substantially by univariate or multivariate SEM approaches. Women who reported use of hormone replacement therapy in the past did not significantly differ in levels of CR across all measurements of CR (Supplementary Table 5).

### 3.3 Reserve estimates and incident MCI

In separate Cox models (Table 2; left panel) adjusting for multiple potential confounders, the observed associations of lower MCI risks with each of the domain-specific measures were stronger with CR estimates from the multivariate first-order correlated reserve SEM and the higher-order SEM model with general reserve structure, as compared to those based on the orthogonal reserve estimates. An increase in each of the five CR estimates from the correlated reserve SEM was significantly inversely related to risk for MCI. Similar associations were estimated when using CR estimates from the higher-order SEM (data not shown). Compared to the domain-specific reserve, the general CR measure exerted the strongest protection against MCI: older women who were one standard deviation above the population mean were 93% (HR = 0.07; 95% CI = 0.04–0.13) less likely to develop incident MCI than those with an average general CR. When all reserve measures were included in the joint Cox models, the strength of the respective associations was attenuated, especially for attention and language reserve, which were no longer associated with MCI risk. Across five specific domains, verbal memory reserve was most strongly associated with reduced risk for MCI. Similar patterns of joint model associations were observed with domain-specific CR measures estimated across univariate, multivariate orthogonal reserve, and multivariate correlated reserve SEMs, as well as the higher-order SEM (data not shown).

**Table 2:** Results of Cox-proportional hazards regression analyses examining the association between reserve variables and incident mild cognitive impairment (number of events = 156)∗

Reserve	Separate model estimates†			Joint model estimates†		
Domain	Coef	HR (95% CI)	*P*	Coef	HR (95% CI)	*P*
Multivariate first-order orthogonal reserve SEM estimates‡						
Attention	−.13	.88 (.67–1.14)	.34	−.08	.92 (.70–1.20)	.53
Verbal memory	−.68	.51 (.38–.68)	<.01	−.70	.50 (.37–.66)	<.01
Figural memory	−.66	.52 (.36–.75)	<.01	−.44	.64 (.49–.84)	<.01
Language	−.39	.68 (.48–.96)	.03	−.21	.81 (.60–1.11)	.19
Spatial	−.24	.79 (.61–1.02)	.07	−.30	.74 (.58–.94)	.02
Multivariate first-order correlated reserve SEM estimates‡						
Attention	−.50	.61 (.48–.77)	<.01	−.09	.91 (.70–1.19)	.49
Verbal memory	−1.00	.37 (.29–.47)	<.01	−.71	.49 (.37–.66)	<.01
Figural memory	−.87	.42 (.34–.52)	<.01	−.43	.65 (.50–.85)	<.01
Language	−.87	.42 (.32–.55)	<.01	−.20	.82(.60–1.12)	.21
Spatial	−.61	.54 (.43–.68)	<.01	−.30	.74 (.58–.94)	.02
Multivariate higher-order SEM estimate of general reserve‡						
General	−2.60	.07 (.04–.13)	<.01	N/A§	N/A	N/A

Abbreviations: MRI, magnetic resonance imaging; SEM, structural equation model.

∗ All models adjust for days between cognitive assessment and MRI, age, education, ethnicity, employment, structural brain neuropathology, region, smoking, alcohol use, depressive symptoms, exercise, hormone assignment, diabetes, cholesterol, hypertension, and cardiovascular disease. All reserve variables scaled to have a mean = 0 and SD = 1. Baseline time was the day of the MRI evaluation.

† Separate models examine each reserve variable separately while joint estimates include all reserve variables in the same model. (General reserve could not be estimated in the joint models).

‡ Reserve variables from first-order uncorrelated reserve as depicted in Fig. 2A, from multivariate first-order correlated factor model as depicted in Fig. 2B, while multivariate higher-order SEM is depicted in Fig. 2C.

§ General reserve could not be estimated in the joint models.

### 3.4 Reserve estimates and incident dementia

Similar to the results from separate Cox models on MCI, the inverse association of dementia risk with each reserve factor was stronger with CR estimates from the multivariate first-order correlated reserve SEM, as compared to the orthogonal reserve estimates. Increased CR in each of the domains of verbal memory, figural memory, language, and spatial ability was each associated with significantly lower dementia risk (Table 3; left panel). Older women who scored one standard deviation above the mean of general CR had 72% lower risk for dementia (HR = 0.28; 95% CI, 0.16–0.52) than those at the population mean, and this inverse association was much stronger than those observed with the domain-specific reserve estimates. When the five first-order reserve measures were jointly modeled, the apparently protective association of CR diminished, with only verbal memory reserve significantly associated with lower dementia risk (*P* = .01).

**Table 3:** Results of Cox-proportional hazards regression analyses examining the association between reserve variables and all-cause dementia (number of events = 104)∗

Reserve	Separate model estimates†			Joint model estimates†			
Domain	Coef	HR (95% CI)	*P*	Coef	HR	95% CI	*P*
Multivariate first-order orthogonal reserve SEM estimates‡							
Attention	.12	1.13 (.82–1.54)	.45	.19	1.21 (.90–1.62)		.20
Verbal memory	−.59	.55 (.38–.81)	<.01	−.52	.59 (.43–.82)		<.01
Figural memory	−.08	.92 (.58–1.46)	.72	−.13	.88 (.64–1.20)		.42
Language	−.46	.63 (.42–.96)	.03	−.35	.71 (.50-.99)		.04
Spatial	−.11	.90 (.66–1.22)	.49	−.15	.86 (.65–1.13)		.28
Multivariate first-order correlated reserve SEM estimates‡							
Attention	−.10	.90 (.69–1.18)	.45	.22	1.25 (.92–1.70)		.15
Verbal memory	−.67	.51 (.38–.69)	<.01	−.52	.59 (.42–.84)		<.01
Figural memory	−.37	.69 (.52–.91)	.01	−.10	.90 (.64–1.28)		.57
Language	−.55	.58 (.42–.79)	<.01	−.34	.71 (.49–1.03)		.07
Spatial	−.29	.75 (.58–.97)	.03	−.14	.87 (.65–1.16)		.34
Multivariate higher-order SEM estimate of general reserve‡							
General	−1.26	.28 (.16–.52)	<.01	N/A§	N/A	N/A	N/A

Abbreviations: MRI, magnetic resonance imaging; SEM, structural equation model.

∗ All models adjust for days between cognitive assessment and MRI, age, education, ethnicity, employment, structural brain neuropathology, region, smoking, alcohol use, depressive symptoms, exercise, hormone assignment, diabetes, cholesterol, hypertension, and cardiovascular disease. All reserve variables scaled to have a mean = 0 and SD = 1. Baseline time was the day of the MRI evaluation.

† Separate models examine each reserve variable separately while joint estimates include all reserve variables in the same model. (General reserve could not be estimated in the joint models.)

‡ Reserve variables from first-order uncorrelated reserve as depicted in Fig. 2A, from multivariate first-order correlated factor model as depicted in Fig. 2B, while multivariate higher-order SEM is depicted in Fig. 2C.

§ General reserve could not be estimated in the joint models.

### 3.5 Sensitivity analyses

After excluding women with incident MCI (Supplementary Table 6) or dementia (Supplementary Table 7) within the first 5 years after the MRI evaluation, the parameter estimates and overall pattern of results were consistent with the main analyses containing the full sample. Next, we systematically varied the amount of error variance specified in our model and examined the extent to which estimates of CR were associated with risk of MCI (Supplementary Table 8) and dementia (Supplementary Table 9). These sensitivity analyses suggest that our findings were robust to the magnitude of error variance specified in our SEM model.

## 4 Discussion

In this longitudinal study conducted in a geographically diverse cohort of older women with both brain MRI data and comprehensive neurocognitive assessments, we quantified the general and multidomain CR measures based on the variance decomposition method. Our study demonstrated that domain-specific reserve constructs were identifiable with moderate correlations among CR measures in attention, verbal memory, figural memory, language, and spatial abilities. CR estimates for verbal memory, figural memory, and spatial abilities showed independent protective effects for MCI, but only verbal memory reserve was associated with reduced risk for dementia. Using a multivariate SEM with higher-order solution, we also estimated a general CR construct, which captured the variance shared across reserve estimates. Although only accounting for a moderate amount of variance in each domain-specific reserve, the general CR measure exerted the strongest protection against both MCI and dementia. To our knowledge, these findings are the first to quantify CR based on residual variance across multiple domains and empirically examine their factor structure and respective associations with subsequent risks of MCI and dementia in a geographically diverse prospective sample of cognitively intact, community-dwelling older women.

The present study extends previous research (see the summary in Supplementary Table 10) beyond the domain of verbal memory CR by showing CR specific to figural memory and visuospatial ability were also protective of incident MCI. Our finding that verbal memory CR was protective of dementia is consistent with previous work using the variance decomposition approach [6], [8]. In addition, our CR measures had good construct validity because all the women in the present study were cognitively intact at the time of cognitive assessment and none of the cognitive tests administered were used in the diagnosis of MCI or dementia. The present study also extends previous work by demonstrating the applicability of this approach in a geographically diverse sample and over a longer follow-up period than previous research. Although the identifiability of general CR with acceptable model fit is consistent with evidence from past studies [6], [14], we added critically important data to the literature by showing that, compared to domain-specific CR measures, general CR was the strongest predictor of lowering the risk for MCI and dementia.

Our study findings have important implications for future research on CR. By drawing an informative sample, we showed the multivariate SEMs, with either correlated reserve or a higher-order solution, fit well with the empirical data from the WHISCA and WHIMS MRI. For both general and domain-specific CR measures identified, construct validity was supported by the results of separate Cox models all showing protection against MCI and dementia (except for attention and language). However, the remarkable difference in the strength of association suggests that the common, general CR construct may better capture the multidimensionality of cognitive resilience that could not be fully represented by single-domain CR factors. These novel observations raise some interesting questions. For instance, are there also domain-specific CR networks that potentially mediate different neuropsychological processes associated with the heterogeneous manifestations of MCI? Do multiple CR domains share a higher-level neural network that operates on a continuum across different stages of neuropathological processes? Future studies with multimodal neuroimaging data are needed to address these important neurobiological questions related to general and domain-specific CR.

Our study also demonstrated the potential usefulness of estimating both domain-specific and general CR. The associations between reserve and risk of MCI and dementia varied by cognitive domain and were attenuated when modeled jointly, thus highlighting the importance of estimating CR across multiple domains. The usefulness of estimating general CR lies in the finding that general CR was a much stronger predictor of incident MCI and dementia than any domain-specific CR estimate. Despite its strong associations with lower risk for cognitive impairment, the general CR variable explained only a moderate proportion of the variance (i.e., less than a third of the variance) in attentional, verbal memory, figural memory, and language CR. Thus, relying solely on a general estimate of CR might neglect some important elements of CR variance that are domain specific. Given the aforementioned advantages and limitations of both domain-specific and general CR, we recommend that researchers estimate both types of CR when using this approach in future research. The differential associations of CR measures across multiple domains with cognitive impairment are consistent with research observing the differentiation of cognitive abilities among individuals who are cognitively intact [15]. These differential associations also point to the possibility that the degree of discrepancy between neuropathology and clinical manifestation may depend on different CR measures or vary across neuropsychological/neuropathological processes. We are currently pursuing analyses to further examine the heterogeneity in CR as well as develop a model to estimate change in CR across multiple domains.

Implementing the residual approach to quantify cognitive reserve has a number of advantages over the sociobehavioral proxy approach. The residual approach is informative as it provides a quantitative estimate of CR that is individual specific and is derived directly from measures of neuropathology and cognitive performance [3]. These estimates are also dynamic and that they potentially allow for examination of factors that increase or deplete CR across age. Given its dynamic nature, the residual approach may be particularly relevant as a potential outcome measure in clinical trials designed to slow cognitive decline and prevent dementia. The residual approach may have important utility in the future for the diagnosis of MCI and dementia by potentially reducing education and racial bias because the estimates of CR derived from the residual approach are independent of these variables. Future research might examine the association between various sociobehavioral indicators and CR measured via the residual method.

Notwithstanding these advantages of the residual approach, there also are limitations of this approach. Like all current approaches to estimating CR, the residual approach is recursive in that estimation of CR is dependent on both the measures of cognitive performance and brain factors. Another limitation includes the fact that the associations between brain variables, cognitive performance, and error are specified by the investigator. In addition, other brain variables that are not included in our model and/or nonlinear associations and interactions between these brain variables and cognitive performance likely play an important role in CR. Because these unmodeled brain variables and associations are not explicitly modeled, they are included in our estimate of CR possibly biasing our estimate of CR.

We recognize further limitations in our study. First, there were a limited number of structural MRI variables included in our model. We included global structural brain indices of whole brain, grey matter, hippocampal, and SVID volumes to be consistent with prior research using the variance decomposition method [6], [8]. Similar to what was reported in prior WHISCA and variance decomposition studies [6], [8], [38], structural MRI variables explained a modest amount of variance in cognitive performance. It is important for future studies to include additional indices of neuropathology that explain additional variance in cognitive performance thereby reducing the residual reserve variance. Second, our study included only women; therefore, results are not generalizable to men. It is important for future work to examine sex differences in CR using a residual approach given the significant sex differences in the prevalence of dementia, and the differential role sex chromosomes, hormones, and other biological factors may play in the etiology of cognitive decline and dementia [18]. Finally, older women included in our informative sample were mostly Caucasian, physically healthy, well educated, with a slightly smaller proportion of participants residing in the south, and all participants had been recruited into a clinical trial of hormone therapy. Thus, our sample was not entirely representative of the general population.

In conclusion, our study finds that general and domain-specific reserve measures are identified with moderate correlation among verbal memory, figural memory, language, and spatial abilities. Across specific domains, verbal memory reserve had the strongest association with MCI and dementia risk. These findings suggest cognitive reserve is a heterogeneous construct with valid quantitative measures identifiable across different neuropsychological processes leading to MCI and dementia.

**Research in context**

1. Systematic review: A literature search (e.g., PubMed, Google Scholar) was conducted to identify literature focusing on measurement of cognitive reserve. From this review, it was unclear if cognitive reserve is best defined as a general or cognitive domain–specific trait and whether general and domain-specific cognitive reserve differentially predicts future cognitive impairment.
2. Interpretation: Variance decomposition estimates of general and domain-specific reserve were identifiable with verbal memory, figural memory, and spatial reserve, each independently associated with lowering mild cognitive impairment risk. Only verbal memory reserve was associated with reduced dementia risk. General reserve had the strongest association with mild cognitive impairment and dementia. Our study results suggest that cognitive reserve is a heterogeneous construct with valid quantitative measures identifiable across different neuropsychological processes associated with mild cognitive impairment and dementia.
3. Future directions: Furthermore, this study highlights the validity of estimating cognitive reserve via the residual approach.
