# Supplementary material for: General and domain-specific cognitive reserve, mild cognitive impairment, and dementia risk in older women
Source: Alzheimers Dement (N Y). 2019 Apr 10;5:118–28. doi: 10.1016/j.trci.2019.02.003 (PMC6461572; doi:10.1016/j.trci.2019.02.003)
Supplement: Supplementary Data [file mmc1.docx]

**Supplemental Data**

General and Domain-Specific Cognitive Reserve, Mild Cognitive Impairment, and Dementia Risk in Older Women

**Supplemental Data Table of contents:**

-Page 3-7: Supplemental methods

-Page 8: Table 1- Comparison of WHISCA participants included vs excluded in analyses

-Page 9: Table 2- SEM model fit statistics

-Page 10: Table 3- Standardized path estimates from the higher-order general reserve model.

-Page 11: Table 4- Correlations between reserve variables derived from SEM models

-Page 12: Table 5- Comparison of estimated level of cognitive reserve by women who had a past history of Hormone replacement therapy vs women who have not had hormone replacement therapy in the past.

-Page 13: Table 6- Results of the first sensitivity analysis examining risk of MCI while excluding women with incident MCI within 5-years after the MRI evaluation

-Page 14: Table 7- Results of the second sensitivity analysis examining risk of dementia while excluding women with incident dementia within 5-years after the MRI evaluation

-Page 15: Table 8- Results of the third sensitivity analysis where the error variance in the variance decomposition model is systematically varied between zero error variance to 20% error variance and the association with MCI.

-Page 16: Table 9- Results of the fourth sensitivity analysis where the error variance in the variance decomposition model is systematically varied between zero error variance to 20% error variance and the association with dementia

-Pages 17-19: Table 10- Summary of prior research utilizing the variance decomposition method to quantify cognitive reserve.

-Page 20: Figure 1- Diagram of the structural equation model to estimate CR while adding an additional pathway from intracranial volume to performance in each cognitive domain.

-Page 21: Table 11- Model fit statistics from the modified structural equation models (SEM) adding an additional pathway from intracranial volume to performance in each cognitive domain as depicted in supplemental figure 1.

-Page 22: Table 12- Results of cox-proportional hazards regression analyses examining the association between reserve variables with performance adjusted for ICV and incident mild cognitive impairment (number of events = 156).

Page 23: Table 13- Results of cox-proportional hazards regression analyses examining the association between reserve variables and all-cause dementia (number of events = 104)^*^

Pages 24-26: Supplemental reference section

**Supplemental Methods**

*Assessment of cognitive performance*

The WHISCA neurocognitive battery measured the following cognitive domains: attention, verbal memory, figural memory, language, and spatial ability. The forwards and backwards digit span from the Wechsler Adult Intelligence Scale [1] were administered to measure attention. The sum number of words successfully recalled across the California Verbal Learning Test [2] (CVLT) list A learning trials and total number of words freely recalled after a long delay were used to measure verbal memory. In WHISCA only three CVLT learning trials were administered instead of the standard five trials. The number of errors on the Benton Visual Retention Test (BVRT) [3] was used to measure figural memory. Letter (F-A-S) [4] and category fluency (vegetables, fruits) [5] were used to measure language ability. The Card Rotations Test [6] was administered to measure spatial ability. All indices were transformed so a higher score represented better performance and z-score standardized based on the initial WHISCA mean and SD. Composite scores for each domain were created by computing the average score across all tests within a domain. Prior research in WHISCA has identified a significant CVLT form effect [7]; therefore, the verbal memory composite score was adjusted for CVLT form using regression analyses.

*Description of the structural MRI assessment*

All of the clinical sites followed the same standardized scan acquisition and processing protocol. A 1.5T scanner was used to acquire T1- and T2- proton density-weighted and fluid attenuated inversion recovery (FLAIR) scans. The structural MRI variables utilized in this study included: total normal brain volume (total brain volume), total grey matter, hippocampal volumes (left and right), total volume of small vessel ischemic diseases (SVID), and intracranial volume. Automated computer-based template warping methods were used to obtain volumetric measurements of total brain volume, total grey matter, and both hippocampal volumes. Total small vessel ischemic diseas was segments via a brain lesion segmentation algorithm. The protocols used in this examination are described in greater detail in previously published studies [8,9,10].

*Variance decomposition approach to quantify reserve*

Structural equation modeling (SEM) was used to decompose the variance in each performance within each cognitive domain explained by neuropathology, demographic factors, and an unmeasured residual which was hypothesized to capture reserve in that respective cognitive domain. We drew upon the approach taken in the original variance decomposition paper [11] as a guide for these analyses. Our model differs from the approach first proposed by Reed et al. [6] in five ways: 1) we estimated a hippocampal volume latent factor which is an estimate of variance in common to right and left hippocampi, 2) we estimated a “total normal brain volume” latent factor which consisted of shared variance between global normal appearing brain volume and global normal appearing grey matter, 3) we regressed each composite estimate of each cognitive domain on age, 4) we regressed each composite of cognitive performance directly onto MRI and demographic variables instead of estimating these association through causal factors representing brain and demographic variables, 5) total grey matter and minority status were allowed to correlated with each other in the model. The rationale for each decision is described next. First, because we were modeling verbal and non-verbal memory we wanted to incorporate estimates of hippocampal volume from both hemispheres. Second, we also wanted to incorporate more comprehensive global estimates of normal appearing brain volume which is why we created our latent factor described in the second deviation. Third, Reed et al. [6] report that their estimates of memory reserve were not correlated with age. Our estimates of reserve were significantly correlated with age when we did not incorporate age in the model. We, therefore, we chose to regress cognitive performance composite onto age to ensure our estimate of reserve was independent of age. Fourth, we did not estimate causal demographic or brain factors because in our models because we are examining performance across multiple domains. Fifth, examination of post-fit indices was conducted to identify parts of the model that should be added or dropped. There was a significant correlation between total grey matter volume and minority status, therefore these variables were allowed to correlate in the model.

In our model, we estimated a hippocampal volume latent factor which was an estimate of the variance in volume common to right and left hippocampi. The normal brain volume latent factor consisted of an estimate of the variance in brain volume in common to hippocampal volumes, normal total grey matter volume, and normal total brain volume. Log-transformed global small-vessel-ischemic disease (SVID) volume was used to quantify abnormal brain volume. Hippocampal volumes, normal brain volume, and SVID were all coded so that a higher score represented more severe neuropathology. All brain volumes were adjusted for age and total intracranial volume. Demographic factors associated with worse cognitive performance including education and ethnicity were also included in the model. Two categorical variables for education were included in the model: (1) having less than a high school education: No=0, Yes = 1 and (2) having a high school education: No =0, Yes =1, with the reference group had at least a college degree. One dichotomous variable was included for ethnic minority status: 0=No, 1=Yes). Each cognitive performance composite score was regressed on the age of the participant at cognitive testing, hippocampal volume, normal brain volume, SVID volume, education, minority status, and reserve factor (residual variance in cognitive performance that is not explained by either neuropathology or demographic factors). A higher reserve score for a participant indicated that the participant’s composite score in the respective cognitive domain was higher than what would be predicted given the individual’s combination of age, neuropathology, and demographic characteristics. The mean of the reserve factor was z-score standardized so the mean is zero with a standard deviation of one. The model also specified that the reserve factor was orthogonal to the neuropathology and demographic factors. The residual variance of the demographic and neuropathology factors were set to zero. To account for error in the estimation of the MRI variables and cognitive performance variables, a residual variance of .10 the total variance was set for each MRI variable and .15 for each domain of cognitive performance.

First, we constructed a univariate SEM approach where each cognitive domain and domain-specific reserve index was modeled separately. This was to ensure that reserve within each domain could be estimated with adequate model fit before moving to multivariate modeling. Next, multivariate model building proceeded in three incremental steps: (1) a first-order multivariate orthogonal reserve SEM approach where all domain-specific reserve factors were simultaneously estimated in the same SEM model and domain-specific reserve factors were not allowed to correlate with each other. (depicted in Figure 2a), a first-order multivariate correlated reserve SEM approach where all domain-specific reserve factors were simultaneously estimated and allowed to correlate with each other (depicted in Figure 2b), and (3) a higher-order reserve multivariate SEM approach where all domain-specific factors were estimated along with estimating a higher order latent variable that we call “general cognitive reserve” (Figure 2c) that captures variance common across all domains. Comparisons of the fit of the three multivariate models allow a formal test to identify the model that best describing the associations between domain-specific reserve variables. Multiple indices were used to evaluate model fit: the comparative fit index (CFI), Tucker Lewis Index (TLI), the root-mean-squared error of approximation (RMSEA) with 90% confidence interval (CI), and the chi-square by degrees of freedom. Standard criteria of CFI > .95, TLI > .95, and RMSEA < .06 was adapted to represent acceptable model fit [12]. All SEM’s were fit with the program MPLUS version 8 [13] and the MPLUS automation package [14] in R [15].

After fitting these models we further examined possible areas of model misfit. We identified that a significant correlation between ICV and performance within each cognitive domain and prior research has identified a significant correlation between larger ICV and better cognitive performance [16]. We fit an exploratory model where performance within each cognitive domain was also regressed on ICV (see supplemental figure 1 for a depiction of this model). We then examined the overall fit of these univariate and multivariate models (see supplemental table 11 for model fit indices). Lastly, we examined the associations between these estimates of CR with incident MCI (see supplemental table 12) and dementia (see supplemental table 13).

| Supplemental Table 1. Comparison of WHISCA participants included vs excluded in analyses | | | | | | | |  |
| --- | --- | --- | --- | --- | --- | --- | --- | --- |
|  | | **Excluded N = 1,332** | | **Included N=972** | |  |  |  |
|  | | **Mean** | **SD** | **Mean** | **SD** | **t** |  |  |
|  | | **or** | **or** | **or** | **Or** | **or** |  |  |
|  | | **N** | **%** | **N** | **%** | **χ^2^** | ***p*** |  |
| Baseline WHISCA age (years) | | 73.77 | 3.83 | 72.86 | 3.64 | 5.75 | <0.01 |  |
| U.S. Region | |  |  |  |  | 12.20 | 0.01 |  |
| Northeast | | 309 | 23.20 | 168 | 17.28 |  |  |  |
| South | | 193 | 14.49 | 150 | 15.43 |  |  |  |
| Midwest | | 489 | 36.71 | 393 | 40.43 |  |  |  |
| West | | 341 | 25.60 | 261 | 26.85 |  |  |  |
| Ethnicity | |  |  |  |  | 15.81 | <0.01 |  |
| African-American | | 106 | 7.69 | 38 | 3.91 |  |  |  |
| Hispanic White | | 16 | 1.20 | 13 | 1.34 |  |  |  |
| White (not of Hispanic) | | 1175 | 88.21 | 896 | 92.18 |  |  |  |
| Other or Missing | | 35 | 2.63 | 25 | 2.57 |  |  |  |
| Education | |  |  |  |  | 8.92 | 0.01 |  |
| Less than high school | | 84 | 6.33 | 35 | 3.60 |  |  |  |
| High school | | 287 | 21.64 | 206 | 21.19 |  |  |  |
| College or more | | 955 | 72.02 | 731 | 75.21 |  |  |  |
| Geriatric Depression Scale | | 1.70 | 2.26 | 1.24 | 1.86 | 5.14 | <0.01 |  |
| Cognitive Performance | |  |  |  |  |  |  |  |
| Attention/Working Memory | |  |  |  |  |  |  |  |
| Digit Span Forward | | 7.35 | 2.02 | 7.56 | 2.09 | -2.51 | 0.01 |  |
| Digit Span Backwards | | 6.53 | 1.97 | 6.85 | 2.06 | -3.87 | <0.01 |  |
| Language | |  |  |  |  |  |  |  |
| Phonemic Fluency | | 38.97 | 12.71 | 40.57 | 12.11 | -3.05 | <0.01 |  |
| Semantic Fluency | | 28.41 | 6.30 | 29.68 | 6.11 | -4.83 | <0.01 |  |
| Visuospatial | |  |  |  |  |  |  |  |
| Card Rotations | | 51.7 | 28.35 | 59.20 | 26.16 | -6.47 | <0.01 |  |
| Visual Episodic Memory | |  |  |  |  |  |  |  |
| Benton Visual Retention | | 7.69 | 4.04 | 6.38 | 3.31 | 8.24 | <0.01 |  |
| Verbal Episodic Memory | |  |  |  |  |  |  |  |
| CVLT total correct trials 1-3 | | 27.64 | 6.55 | 29.98 | 5.86 | -8.87 | <0.01 |  |
| CVLT long delay free recall | | 8.64 | 3.25 | 9.77 | 2.69 | -8.85 | <0.01 |  |
|  | |  |  |  |  |  |  |  |
| Supplemental Table 2. SEM Model fit statistics | | | | | | | | |
| Reserve domain | 𝜒2 | df | CFI | TLI | RMSEA | *90% CI RMSEA* | | |
| *Univariate SEM* |  |  |  |  |  |  | | |
| Attention | 48.71 | 23 | .99 | .98 | .03 | [.02 - .05] | | |
| Verbal memory | 67.20 | 23 | .99 | .97 | .04 | [.03 - .06] | | |
| Visual memory | 55.54 | 23 | .99 | .98 | .04 | [.02 - .05] | | |
| Language | 50.35 | 23 | .99 | .98 | .04 | [.02 - .05] | | |
| Visuospatial | 70.10 | 23 | .99 | .97 | .05 | [.03 - .06] | | |
| *Multivariate SEM* |  |  |  |  |  |  | | |
| First-order orthogonal reserve | 244.57 | 45 | .96 | .90 | .07 | [.06 - .08] | | |
| First-order correlated reserve | 128.47 | 35 | .98 | .94 | .05 | [.04 - .06] | | |
| Higher-order general reserve | 163.92 | 40 | .97 | .93 | .056 | [.05 - .07] | | |
| CFI = Comparative fit index; RMSEA= root mean square error of approximation; TLI = Tucker-Lewis Index  first-order orthogonal reserve model corresponds to figure 2a  first-order correlated reserve model corresponds to figure 2b  higher-order general reserve model corresponds to figure 2c | | | | | | | | |

| Supplemental Table 3. Standardized path estimates from the higher-order general reserve model.^*^ | | | | | |
| --- | --- | --- | --- | --- | --- |
| Model component: | | | | | |
| *Structural MRI variables, age, and demographics on cognitive performance* | | | | | |
|  | | | | | |
|  | Attention | Verbal memory | Figural memory | Language | Spatial |
| Hippocampal volume | -0.01  [-0.10 ; 0.10] | -0.12  [-0.21 ; -0.02] | -0.08  [-0.17 ; 0.04] | -0.01  [-0.10 ; 0.09] | 0.01  [-0.10 ; 0.12] |
| Normal brain volume | -0.07  [-0.18 ; 0.03] | -0.01  [-0.11 ; 0.10] | -0.07  [-0.22 ; 0.02] | -0.09  [-0.19 ; 0.01] | -0.19  [-0.32 ; -0.06] |
| Abnormal brain volume | 0.05  [-0.03 ; 0.12] | -0.08  [-0.16 ; -0.01] | -0.07  [-0.13 ; 0.01] | -0.05  [-0.13 ; 0.02] | -0.03  [-0.10 ; 0.04] |
| Age | -0.09  [-0.15 ; -0.03] | -0.17  [-0.23 ; -0.11] | -0.27  [-0.32 ; -0.21] | -0.13  [-0.19 ; -0.07] | -0.27  [-0.32 ; -0.21] |
| Minority status | -0.12  [-0.18 ; -0.05] | -0.11  [-0.17 ; -0.05] | -0.18  [-0.24 ; -0.12] | -0.10  [-0.17; -0.04] | -0.14  [-0.20 ; -0.08] |
| Hs education | -0.08  [-0.14 ; -0.02] | -0.08  [-0.14 ; -0.02] | -0.12  [-0.18 ; -0.06] | -0.09  [-0.15 ; -0.03] | 0.02  [-0.04 ; 0.08] |
| Less than high school education | -0.08  [-0.14 ; -0.02] | -0.08  [-0.14 ; -0.02] | -0.13  [-0.19 ; -0.08] | -0.12  [-0.18 ; -0.06] | -0.03  [-0.09 ; 0.03] |
| Model component: |  |  |  |  |  |
| *General reserve factor to each domain cognitive reserve factor* | | | | | |
|  | Attention | Verbal memory | Figural memory | Language | Spatial |
| General reserve | 0.45  [0.37 ; 0.53] | 0.54  [0.47 ; 0.62] | 0.69  [0.61 ; 0.76] | 0.57  [0.49 ; 0.65] | 0.48  [0.41 ; 0.56] |

^*^ In order to simplify the presentation of the standardized path estimates not all path estimates are presented here. Specifically, path estimates of intracranial volume and age on MRI variables are not presented here. Path estimates of latent hippocampal volume on right and left hippocampal volume are not presented as are path estimates of latent normal brain volume on total brain volume and total normal grey matter are not provided. These parameter estimates are available upon request.

| Supplemental Table 4. Correlations between reserve variables derived from SEM models | | | | | | | |
| --- | --- | --- | --- | --- | --- | --- | --- |
|  | Attention | | Verbal memory | Figural memory | Language | Spatial | General CR |
| *Univariate SEM estimates* | | |  |  |  |  |  |
| Attention | 1 |  | |  |  |  |  |
| Verbal memory | 0.17 | 1 | |  |  |  |  |
| Figural memory | 0.26 | 0.32 | | 1 |  |  |  |
| Language | 0.27 | 0.32 | | 0.27 | 1 |  |  |
| Spatial | 0.15 | 0.16 | | 0.34 | 0.22 | 1 |  |
| *Multivariate first-order correlated factors model* | | | | | | | |
| Attention | 1 |  | |  |  |  |  |
| Verbal memory | 0.24 | 1 | |  |  |  |  |
| Figural memory | 0.36 | 0.44 | | 1 |  |  |  |
| Language | 0.37 | 0.43 | | 0.38 | 1 |  |  |
| Spatial | 0.20 | 0.23 | | 0.47 | 0.30 | 1 |  |
| *Multivariate higher-order general reserve model* | | | | | | | |
| Attention | 1 |  | |  |  |  |  |
| Verbal memory | 0.25 | 1 | |  |  |  |  |
| Figural memory | 0.36 | 0.43 | | 1 |  |  |  |
| Language | 0.34 | 0.41 | | 0.40 | 1 |  |  |
| Spatial | 0.22 | 0.25 | | 0.44 | 0.31 | 1 |  |
| General CR | 0.57 | 0.68 | | 0.84 | 0.71 | 0.61 | 1 |
| -All correlations are significant at *p* < 0.01 | | | | | | | |
| -Univariate SEM reserve estimates derived via separate SEM model for each factor | | | | | | | |
| -Reserve estimates from first-order correlated factors multivariate SEM | | | | | | | |
| -Reserve estimates from multivariate SEM with higher-order CR derived from multivariate model estimating reserve across all domains as depicted in figure 2c. | | | | | | | |

| Supplemental Table 5. Comparison of estimated level of cognitive reserve by women who had a past history of Hormone replacement therapy vs women who have not had hormone replacement therapy in the past. | | | | | | |
| --- | --- | --- | --- | --- | --- | --- |
|  | **Hormone use ever** | | | |  |  |
|  | **Yes**  **N = 461** | | **No**  **N=511** | |  |  |
|  | **Mean** | **SD** | **Mean** | **SD** | **t** | **p** |
| *Multivariate first-order orthogonal reserve SEM estimates ^‡^* | | | | | |  |
| Attention | -.01 | .63 | .01 | .61 | -.39 | .70 |
| Verbal memory | <.01 | .55 | <.01 | .56 | .027 | .99 |
| Figural memory | -.02 | .45 | .02 | .47 | 1.51 | .13 |
| Language | .01 | .50 | -.01 | .52 | -.78 | .44 |
| Spatial | .01 | .63 | -.01 | .62 | -.55 | .58 |
| *Multivariate first-order correlated reserve SEM estimates ^‡^* | | | | | |  |
| Attention | .01 | .73 | <.01 | .74 | -.23 | .82 |
| Verbal memory | <.01 | .72 | .01 | .72 | .17 | .87 |
| Figural memory | -.03 | .76 | .03 | .78 | 1.07 | .28 |
| Language | .01 | .66 | -.01 | .71 | -.48 | .63 |
| Spatial | .01 | .76 | <.01 | .78 | -.31 | .76 |
| *Multivariate higher-order SEM estimate of General reserve ^‡^* | | | | | |  |
| General | .00 | .33 | .00 | .34 | .27 | .79 |
| first-order orthogonal reserve model corresponds to figure 2a  first-order correlated reserve model corresponds to figure 2b  higher-order general reserve model corresponds to figure 2c | | | | | | |

| Supplemental Table 6. Results of the first sensitivity analysis excluding women with incident MCI within 5-years after the MRI evaluation (number excluded = 43). The parameters presented are from the Cox-proportional hazards regression analyses examining the association between reserve variables and mild cognitive impairment excluding women who developed MCI within five years of the MRI evaluation. (number of events = 113) ^*^ | | | | | | | | |
| --- | --- | --- | --- | --- | --- | --- | --- | --- |
| Reserve | Separate model estimates ^†^ | | | | Joint model estimates ^†^ | | | |
| domain | coef | HR | 95% CI | *p* | coef | HR | 95% CI | *p* |
| *Multivariate first-order orthogonal reserve SEM estimates ^‡^* | | | | | | | | |
| Attention | -.29 | .75 | (.55 – 1.03) | .07 | -.30 | .74 | (.54 – 1.01) | .06 |
| Verbal memory | -.56 | .57 | (.40 - .83) | <.01 | -.66 | .52 | (.37 - .73) | <.01 |
| Figural memory | -.48 | .62 | (.39 - .97) | .04 | -.46 | .63 | (.47 - .85) | <.01 |
| Language | -.36 | .70 | (.47 – 1.05) | .08 | -.28 | .76 | (.53 – 1.08) | .12 |
| Spatial | -.21 | .81 | (.61 – 1.08) | .15 | -.33 | .72 | (.55 - .94) | .02 |
| *Multivariate first-order correlated reserve SEM estimates ^‡^* | | | | | | | | |
| Attention | -.58 | .56 | (.42 – .74) | <.01 | -.26 | .77 | (.56 - 1.06) | .11 |
| Verbal memory | -.92 | .40 | (.29 – .54) | <.01 | -.64 | .53 | (.37 - .76) | <.01 |
| Figural memory | -.83 | .44 | (.33 – .58) | <.01 | -.42 | .66 | (.47 - .91) | .01 |
| Language | -.82 | .44 | (.31 – .62) | <.01 | -.22 | .80 | (.55 – 1.17) | .26 |
| Spatial | -.52 | .59 | (.46 – .77) | <.01 | -.30 | .74 | (.56 - .98) | .04 |
| *Multivariate higher-order SEM estimate of General reserve ^‡^* | | | | | | | | |
| General | -2.71 | .07 | (.03 – .14) | <.01 | N/A^§^ | N/A | N/A | N/A |
| ^*^ All models adjust for time between the MRI and cognitive evaluation, age, education, ethnicity, employment, structural brain neuropathology, region, smoking, alcohol use, depressive symptoms, exercise, hormone assignment, diabetes, cholesterol, hypertension, and cardiovascular disease. All reserve variables scaled to have a mean = 0 and SD = 1.  Baseline time was the day of the MRI evaluation. | | | | | | | | |
| ^†^ Separate models examine each reserve variable separately while joint estimates include all reserve variables in the same model. (General reserve could not be estimated in the joint models) | | | | | | | | |
| *^‡^* Reserve variables from first-order uncorrelated reserve as depicted in figure 2a, from multivariate first-order correlated factor model as depicted in figure 2b, while multivariate higher-order SEM is depicted in figure 2c. | | | | | | | | |
| *^§^* General reserve could not be estimated in the joint models | | | | | | | | |

| Supplemental Table 7. Results of the first sensitivity analysis excluding women with incident dementia within 5-years after the MRI evaluation (number excluded = 25). The parameters presented are from the Cox-proportional hazards regression analyses examining the association between reserve variables and dementia excluding women who developed dementia within five years of the MRI evaluation. (number of events = 88) ^*^ | | | | | | | | |
| --- | --- | --- | --- | --- | --- | --- | --- | --- |
| Reserve | Separate model estimates ^†^ | | | | Joint model estimates ^†^ | | | |
| domain | coef | HR | 95% CI | *p* | coef | HR | 95% CI | *p* |
| *Multivariate first-order orthogonal reserve SEM estimates ^‡^* | | | | | | | | |
| Attention | .11 | 1.12 | (.80 - 1.57) | .51 | .19 | 1.21 | (.88 - 1.65) | .24 |
| Verbal memory | -.49 | .61 | (.40 - .93) | .02 | -.44 | .64 | (.45 - .93) | .02 |
| Figural memory | .01 | 1.01 | (.61 - 1.69) | .96 | -.05 | .95 | (.67 - 1.34) | .76 |
| Language | -.49 | .61 | (.38 - .97) | .04 | -.41 | .66 | (.45 - .97) | .04 |
| Spatial | -.18 | .84 | (.60 - 1.16) | .29 | -.22 | .80 | (.60 - 1.08) | .15 |
| *Multivariate first-order correlated reserve SEM estimates ^‡^* | | | | | | | | |
| Attention | -.09 | .91 | (.69 - 1.22) | .55 | .22 | 1.25 | (.90 - 1.74) | .19 |
| Verbal memory | -.59 | .55 | (.39 - .78) | <.01 | -.43 | .65 | (.44 - .96) | .03 |
| Figural memory | -.32 | .73 | (.54 - .99) | .04 | -.02 | .98 | (.67 – 1.44) | .92 |
| Language | -.56 | .57 | (.40 - .81) | <.01 | -.41 | .66 | (.44- 1.01) | .05 |
| Spatial | -.32 | .73 | (.55 - .96) | .03 | -.22 | .80 | (.59 - 1.11) | .18 |
| *Multivariate higher-order SEM estimate of General reserve ^‡^* | | | | | | | | |
| General | -1.18 | .31 | (.15 - .61) | <.01 | N/A^§^ | N/A | N/A | N/A |
| ^*^ All models adjust for time between the MRI and cognitive evaluation, age, education, ethnicity, employment, structural brain neuropathology, region, smoking, alcohol use, depressive symptoms, exercise, hormone assignment, diabetes, cholesterol, hypertension, and cardiovascular disease. All reserve variables scaled to have a mean = 0 and SD = 1.  Baseline time was the day of the MRI evaluation. | | | | | | | | |
| ^†^ Separate models examine each reserve variable separately while joint estimates include all reserve variables in the same model. (General reserve could not be estimated in the joint models) | | | | | | | | |
| *^‡^* Reserve variables from first-order uncorrelated reserve as depicted in figure 2a, from multivariate first-order correlated factor model as depicted in figure 2b, while multivariate higher-order SEM is depicted in figure 2c. | | | | | | | | |
| *^§^* General reserve could not be estimated in the joint models | | | | | | | | |

| Supplemental Table 8. Results of the third sensitivity analysis where the error variance in the variance decomposition model is systematically varied between zero error variance to 20% error variance. The parameters presented are from the Cox-proportional hazards regression analyses examining the association between the first-order correlated and general cognitive reserve variables with MCI risk. (number of events = 156) ^*^ | | | | | | | | | | |
| --- | --- | --- | --- | --- | --- | --- | --- | --- | --- | --- |
|  | Separate model estimates ^†^  HR (p) | | | | | Joint model estimates ^†^  HR (p) | | | | |
| Error variance specified in SEM model | 0% | 5% | 10% | 15% | *20%* | 0% | 5% | 10% | 15% | 20% |
| *Multivariate first-order correlated reserve SEM estimates ^‡^* | | | | | | | | | | |
| Attention | .69  (<.01) | .66  (<.01) | .64  (<.01) | .61  (<.01) | .58  (<.01) | .90 (.33) | .90  (.38) | .90  (.44) | .92  (.53) | .92  (.57) |
| Verbal memory | .46  (<.01) | .43  (<.01) | .40  (<.01) | .37  (<.01) | .33  (<.01) | .54 (<.01) | .53  (<.01) | .51  (<.01) | .50  (<.01) | .47  (<.01) |
| Figural memory | .52  (<.01) | .49  (<.01) | .45  (<.01) | .42  (<.01) | .38  (<.01) | .68  (<.01) | .66  (<.01) | .66  (<.01) | .64  (<.01) | .63  (<.01) |
| Language | .52  (<.01) | .49  (<.01) | .46  (<.01) | .42  (<.01) | .38  (<.01) | .80  (.08) | .81  (.11) | .81  (.15) | .81  (.19) | .83  (.29) |
| Spatial | .64  (<.01) | .61  (<.01) | .57  (<.01) | .54  (<.01) | .50  (<.01) | .76  (<.01) | .76  (<.01) | .75  (.01) | .74  (.02) | .73  (.03) |
| *Multivariate higher-order SEM estimate of General reserve ^‡^* | | | | | | | | | | |
| General | .07  (<.01) | .07  (<.01) | .07  (<.01) | .07  (<.01) | .07  (<.01) | N/A^§^ | N/A | N/A | N/A | N/A |
| ^*^ All models adjust for time between the MRI and cognitive evaluation, age, education, ethnicity, employment, structural brain neuropathology, region, smoking, alcohol use, depressive symptoms, exercise, hormone assignment, diabetes, cholesterol, hypertension, and cardiovascular disease. All reserve variables scaled to have a mean = 0 and SD = 1.  Baseline time was the day of the MRI evaluation. | | | | | | | | | | |
| ^†^ Separate models examine each reserve variable separately while joint estimates include all reserve variables in the same model. (General reserve could not be estimated in the joint models) | | | | | | | | | | |
| *^‡^* Reserve variables from multivariate first-order correlated factor model as depicted in figure 2b, while multivariate higher-order SEM is depicted in figure 2c. | | | | | | | | | | |
| *^§^* General reserve could not be estimated in the joint models | | | | | | | | | | |

| Supplemental Table 9. Results of the fourth sensitivity analysis where the error variance in the variance decomposition model is systematically varied between zero error variance to 20% error variance. The parameters presented are from the Cox-proportional hazards regression analyses examining the association between the first-order correlated and general cognitive reserve variables with dementia risk. (number of events = 104) ^*^ | | | | | | | | | | |
| --- | --- | --- | --- | --- | --- | --- | --- | --- | --- | --- |
|  | Separate model estimates ^†^  HR (p) | | | | | Joint model estimates ^†^  HR (p) | | | | |
| Error variance specified in SEM model | 0% | 5% | 10% | 15% | *20%* | 0% | 5% | 10% | 15% | 20% |
| *Multivariate first-order correlated reserve SEM estimates ^‡^* | | | | | | | | | | |
| Attention | .94  (.60) | .93  (.55) | .91  (.50) | .90  (.45) | .89  (.40) | 1.17  (.19) | 1.20  (.17) | 1.22  (.16) | 1.21  (.20) | 1.28  (.14) |
| Verbal memory | .58  (<.01) | .56  (<.01) | .54  (<.01) | .51  (<.01) | .49  (<.01) | .64  (<.01) | .63  (<.01) | .61  (<.01) | .59  (<.01) | .57  (<.01) |
| Figural memory | .76  (.02) | .74  (.02) | .72  (.01) | .69  (.01) | .65  (<.01) | .90  (.42) | .90  (.46) | .90  (.52) | .88  (.42) | .91  (.64) |
| Language | .64  (<.01) | .63  (<.01) | .60  (<.01) | .58  (<.01) | .55  (<.01) | .75  (.04) | .73  (.05 | .73  (.06) | .71  (.04) | .70  (.09) |
| Spatial | .80  (.05) | .79  (.04) | .77  (.04) | .75  (.03) | .72  (.02) | .88  (.27) | .88  (.29) | .87  (.32) | .86  (.28) | .86  (.38) |
| *Multivariate higher-order SEM estimate of General reserve ^‡^* | | | | | | | | | | |
| General | .28  (<.01) | .28  (<.01) | .28  (<.01) | .28  (<.01) | .28  (<.01) | N/A^§^ | N/A | N/A | N/A | N/A |
| ^*^ All models adjust for time between the MRI and cognitive evaluation, age, education, ethnicity, employment, structural brain neuropathology, region, smoking, alcohol use, depressive symptoms, exercise, hormone assignment, diabetes, cholesterol, hypertension, and cardiovascular disease. All reserve variables scaled to have a mean = 0 and SD = 1.  Baseline time was the day of the MRI evaluation. | | | | | | | | | | |
| ^†^ Separate models examine each reserve variable separately while joint estimates include all reserve variables in the same model. (General reserve could not be estimated in the joint models) | | | | | | | | | | |
| *^‡^* Reserve variables from multivariate first-order correlated factor model as depicted in figure 2b, while multivariate higher-order SEM is depicted in figure 2c. | | | | | | | | | | |
| *^§^* General reserve could not be estimated in the joint models | | | | | | | | | | |

| Supplemental Table 10. Summary of prior research utilizing the variance decomposition method to quantify cognitive reserve. | | | | | | |
| --- | --- | --- | --- | --- | --- | --- |
| **References** | **Population Characteristics**:  Clinical population vs. community-dwelling populations  Single Center/Local samples vs. multi-center/geographically-diverse samples | **CR Domain(s) Studied**: | **Cognitive Status at the Time of CR Measure**: including those with cognitive impairment vs. all cognitively intact vs. | **General CR Defined**: Yes/No.  If yes, how it was defined | **Cognitive Endpoints to Validate CR**: cognitive decline vs. MCI vs. dementia; whether potential confounders (and what) were adjusted | **Characterization of Neuropathologies** |
| Reed et al.[11] | 305 participants >60 years old (mean age: 74.5±7.2 years) recruited through community outreach (74%) and the remaining participants (26%) were undergoing clinical evaluation at the UC Davis Alzheimer’s Disease Centre | Episodic memory (primary); semantic memory (secondary) | Cognitively intact, mild cognitive impairment, and demented cases | No | CR 🡪 global clinical status (normal; MCI; dementia); CR 🡪 reading ability; CR 🡪 conversion from MCI to dementia; CR 🡪 longitudinal change in Executive Function  Confounders: demographics (years of formal education, sex [female as reference], Hispanic or African American ethnicity [Caucasian as reference]), neuropathology | Brain matter volume and hippocampal volume (adjusted for intracranial volume); white matter hyperintensities |
| Reed et al.[17] | 652 autopsied cases from two community-based studies: the Rush Memory (mean age: 75 years) and Aging Project (mean age: 81 years) and the Religious Orders Study | Episodic memory; semantic memory; fluency; working memory; perceptual organization; perceptual speed | Cognitively intact, mild cognitive impairment, and demented cases | Yes, second-order factor formed by the six domain-specific reserve terms and it was the primary measure of reserve used in the study | No cognitive endpoints  Confounders: socioeconomic status, education, leisure cognitive activities at age 40 and at baseline | Neuritic plaques, diffuse plaques, neocortical neurofibrillary tangles, medial temporal neurofibrillary tangles, brain weight (adjusted for gender and height), Lewy bodies, chronic microscopic infarctions, and macroinfarcts |
| Zahodne et al.[18] | 703 participants of the Washington Heights/Hamilton Heights Inwood Columbia Aging Project, a community-based sample of older adults (mean age: 80.1±5.5 years) | Memory | Cognitively intact, amnestic MCI and non-amnestic MCI | No | CR 🡪 MCI status; CR 🡪 reading ability; CR 🡪 dementia conversion regardless of baseline cognitive status (MCI or no MCI); CR 🡪 longitudinal changes in language (composite of naming, letter fluency, animal fluency, verbal abstraction, repetition, and comprehension)  Confounders: demographics (age, education, sex, race/ethnicity); neuropathology; baseline neuropsychological scores | Brain matter volume and hippocampal volume (adjusted for intracranial volume); white matter hyperintensities |
| Zahodne et al.[19] | 244 participants of the Washington Heights/Hamilton Heights Inwood Columbia Aging Project, a community-based sample of older adults (mean age: 79.4±5.2 years) | Memory | Cognitively intact, amnestic MCI and non-amnestic MCI | No | CR 🡪 incident dementia; CR 🡪 longitudinal changes in language (composite of naming, letter fluency, animal fluency, verbal abstraction, repetition, and comprehension)  Confounders: demographics (age, years of education, sex, race, ethnicity); neuropathology; baseline neuropsychological scores | Total grey matter volume and hippocampal volume (adjusted for intracranial volume); white matter hyperintensities |
| Habeck et al.[20] | 368 community-dwelling adults aged 20-80 years old (mean age: 48.9±18.0 years) | Episodic memory; reasoning ability; perceptual speed; vocabulary | Cognitively intact | Yes, averaged all four CR scores to come up with a single generalized CR score (validated this approach by performing PCA to determine if they could consider the four CR measures as a single underlying construct) | General CR 🡪 verbal intelligence; general CR 🡪 education; four cognitive domains  Confounders: neuropathology; age | Grey matter volume and thickness; fraction anisotropy |

| Supplemental Table 11. SEM Model fit statistics from CR models with cognitive performance regressed on ICV as depicted in supplemental figure 1. | | | | | | |
| --- | --- | --- | --- | --- | --- | --- |
| Reserve domain | 𝜒2 | df | CFI | TLI | RMSEA | *90% CI RMSEA* |
| *Univariate SEM* |  |  |  |  |  |  |
| Attention | 40.29 | 22 | 1.00 | .99 | .03 | [.01 - .04] |
| Verbal memory | 57.23 | 22 | .99 | .98 | .04 | [.03 - .05] |
| Visual memory | 53.36 | 22 | .99 | .98 | .04 | [.02 - .05] |
| Language | 38.39 | 22 | 1.00 | .99 | .03 | [.01 - .04] |
| Visuospatial | 54.29 | 22 | .99 | .98 | .04 | [.03 - .05] |
| *Multivariate SEM* |  |  |  |  |  |  |
| First-order orthogonal reserve | 192.59 | 40 | .97 | .91 | .06 | [.05 - .07] |
| First-order correlated reserve | 76.58 | 30 | .99 | .96 | .04 | [.03 - .05] |
| Higher-order general reserve | 111.53 | 35 | .98 | .95 | .05 | [.04 - .06] |
| CFI = Comparative fit index; RMSEA= root mean square error of approximation; TLI = Tucker-Lewis Index  first-order orthogonal reserve model corresponds to supplemental figure 1a  first-order correlated reserve model corresponds to supplemental figure 1b  higher-order general reserve model corresponds to supplemental figure 1c | | | | | | |

| Supplemental Table 12. Results of cox-proportional hazards regression analyses examining the association between reserve variables with performance adjusted for ICV and incident mild cognitive impairment (number of events = 156) ^*^ | | | | | | | | |
| --- | --- | --- | --- | --- | --- | --- | --- | --- |
| Reserve | Separate model estimates ^†^ | | | | Joint model estimates ^†^ | | | |
| domain | coef | HR | 95% CI | *p* | coef | HR | 95% CI | *p* |
| *Multivariate first-order orthogonal reserve SEM estimates ^‡^* | | | | | | | | |
| Attention | -.12 | .89 | (.68 - 1.15) | .37 | -.08 | .92 | (.71 - 1.21) | .56 |
| Verbal memory | -.72 | .49 | (.36 - .66) | <.01 | -.73 | .48 | (.36 - .64) | <.01 |
| Figural memory | -.64 | .53 | (.36 - .76) | <.01 | -.44 | .64 | (.49 - .84) | <.01 |
| Language | -.36 | .70 | (.49 - .98) | .04 | -.18 | .83 | (.61 - 1.14) | .25 |
| Spatial | -.22 | .80 | (.63 - 1.03) | .08 | -.28 | .75 | (.59 - .96) | .02 |
| *Multivariate first-order correlated reserve SEM estimates ^‡^* | | | | | | | | |
| Attention | -.49 | .61 | (.48 - .78) | <.01 | -.09 | .91 | (.70 - 1.20) | .53 |
| Verbal memory | -1.03 | .36 | (.28 - .46) | <.01 | -.73 | .48 | (.36 - .64) | <.01 |
| Figural memory | -.87 | .42 | (.34 - .52) | <.01 | -.44 | .64 | (.50 - .84) | <.01 |
| Language | -.86 | .42 | (.32 - .56) | <.01 | -.18 | .84 | (.61 - 1.14) | .26 |
| Spatial | -.60 | .55 | (.44 - .69) | <.01 | -.29 | .75 | (.59 - .96) | .02 |
| *Multivariate higher-order SEM estimate of General reserve ^‡^* | | | | | | | | |
| General | -2.48 | .08 | (.05 - .14) | <.01 | N/A^§^ | N/A | N/A | N/A |
| ^*^ All models adjust for days between cognitive assessment and MRI, age, education, ethnicity, employment, intracranial volume, structural brain neuropathology, region, smoking, alcohol use, depressive symptoms, exercise, hormone assignment, diabetes, cholesterol, hypertension, and cardiovascular disease. All reserve variables scaled to have a mean = 0 and SD = 1.  Baseline time was the day of the MRI evaluation. | | | | | | | | |
| ^†^ Separate models examine each reserve variable separately while joint estimates include all reserve variables in the same model. (General reserve could not be estimated in the joint models) | | | | | | | | |
| *^‡^* Reserve variables from first-order uncorrelated reserve as depicted in supplemental figure 1a, from multivariate first-order correlated factor model as depicted in supplemental figure 1b, while multivariate higher-order SEM is depicted in supplemental figure 1c. | | | | | | | | |
| *^§^* General reserve could not be estimated in the joint models | | | | | | | | |

| Supplemental Table 13. Results of cox-proportional hazards regression analyses examining the association between reserve variables and all-cause dementia (number of events = 104)^*^ | | | | | | | | |
| --- | --- | --- | --- | --- | --- | --- | --- | --- |
| Reserve | Separate model estimates^†^ | | | | Joint model estimates^†^ | | | |
| domain | coef | HR | 95% CI | *p* | coef | HR | 95% CI | *p* |
| *Multivariate first-order orthogonal reserve SEM estimates ^‡^* | | | | | | | | |
| Attention | .11 | 1.12 | (.82 - 1.52) | .49 | .18 | 1.20 | (.89 - 1.60) | .24 |
| Verbal memory | -.57 | .57 | (.38 - .84) | <.01 | -.49 | .61 | (.44 - .85) | <.01 |
| Figural memory | -.08 | .92 | (.59 - 1.46) | .73 | -.13 | .88 | (.65 - 1.20) | .42 |
| Language | -.48 | .62 | (.41 - .95) | .03 | -.37 | .69 | (.49 - .97) | .04 |
| Spatial | -.12 | .89 | (.65 - 1.20) | .43 | -.17 | .84 | (.64 - 1.11) | .23 |
| *Multivariate first-order correlated reserve SEM estimates ^‡^* | | | | | | | | |
| Attention | -.11 | .90 | (.68 - 1.16) | .40 | .21 | 1.23 | (.91 - 1.68) | .17 |
| Verbal memory | -.66 | .52 | (.38 - .70) | <.01 | -.48 | .62 | (.43 - .88) | .01 |
| Figural memory | -.38 | .68 | (.52 - .90) | .01 | -.10 | .90 | (.64 - 1.28) | .57 |
| Language | -.58 | .56 | (.41 - .77) | <.01 | -.36 | .70 | (.48 - 1.01) | .06 |
| Spatial | -.31 | .73 | (.56 .96) | .02 | -.16 | .85 | (.64 - 1.14) | .29 |
| *Multivariate higher-order SEM estimate of General reserve ^‡^* | | | | | | | | |
| General | -1.26 | .28 | (.16 - .52) | <.01 | N/A^§^ | N/A | N/A | N/A |
| ^*^ All models adjust for days between cognitive assessment and MRI, age, education, ethnicity, employment,intracranial volume, structural brain neuropathology, region, smoking, alcohol use, depressive symptoms, exercise, hormone assignment, diabetes, cholesterol, hypertension, and cardiovascular disease. All reserve variables scaled to have a mean = 0 and SD = 1.  Baseline time was the day of the MRI evaluation. | | | | | | | | |
| ^†^ Separate models examine each reserve variable separately while joint estimates include all reserve variables in the same model. (General reserve could not be estimated in the joint models) | | | | | | | | |
| *^‡^* Reserve variables from first-order uncorrelated reserve as depicted in supplemental figure 1a, from multivariate first-order correlated factor model as depicted in supplemental figure 1b, while multivariate higher-order SEM is depicted in supplemental figure 1c. | | | | | | | | |
| *^§^* General reserve could not be estimated in the joint models | | | | | | | | |
|  | | | | | | | | |

**Supplemental References**

[1] Wechsler D. Weschler Adult Intelligence Scale-Revised. Psychological Corporation; 1981.

[2] Delis DC, Kramer JH, Kaplan E, Ober BA. California Verbal Learning Test- Research Edition. New York: The Psychological Corporation; 1987.

[3] Benton A. Revised Visual Retention Test. New York: Psychological Corporation; 1974.

[4] Benton AL. Differential behavioral effects in frontal lobe disease. Neuropsychologia 1968;6:53–60.

[5] Newcombe F. Missle wounds of the brain. A study of psychological deficits. London: Oxford University Press; 1969.

[6] Ekstrom RB, French JW, Harman HH. Manual for Kit of factor-referenced cognitive tests. Princeton: Educational Testing Service; 1976.

[7] Resnick SM, Espeland MA, An Y, Maki PM, Coker LH, Jackson R, et al. Effects of conjugated equine estrogens on cognition and affect in postmenopausal women with prior hysterectomy. J Clin Endocrinol Metab 2009;94:4152–61. doi:10.1210/jc.2009-1340.

[8] Resnick SM, Espeland MA, An Y, Maki PM, Coker LH, Jackson R, et al. Effects of conjugated equine estrogens on cognition and affect in postmenopausal women with prior hysterectomy. J Clin Endocrinol Metab 2009;94:4152–61. doi:10.1210/jc.2009-1340.

[9] Resnick SM, Espeland MA, Jaramillo SA, Hirsch C, Stefanick ML, Murray AM, et al. Postmenopausal hormone therapy and regional brain volumes: The WHIMS-MRI study. Neurology 2009; 72: 135-42. doi: 10.1212/01.wnl.0000339037.76336.cf

[10] Coker LH, Hogan PE, Bryan NR, Kuller LH, Margolis KL, Bettermann K, et al. Postmenopausal hormone therapy and subclinical cerebrovascular disease: The WHIMS-MRI study. Neruology 2009; 72: 125-34. doi:10.1212/01.wnl.0000339036.88842.9e

[11] Reed B.R., Mungas D., Farias S.T., Harvey D., Beckett L., Widaman K., et al. Measuring cognitive reserve based on the decomposition of episodic memory variance. Brain J Neurol. 2010; 133: 2196–209. doi:10.1093/brain/awq154.

[12] Byrne BM. Factor analytic models: viewing the structure of an assessment instrument from three perspectives. J Pers Assess 2005;85:17–32. doi:10.1207/s15327752jpa8501_02.

[13] Muthen LK, Muthen BO. MPLUS: Statistical analysis with latent variables User’s Guide. Eigth Edition. Los Angeles, CA: Muthen & Muthen; 1998.

[14] Hallquist MN, Wiley JF. MplusAutomation: An R Package for Facilitating Large-Scale Latent Variable Analyses in Mplus. Struct Equ Model Multidiscip J 2018; 25: 621–38. doi:10.1080/10705511.2017.1402334.

[15] R: A language and environment for statistical computing. Vienna, Austria: R Foundation for Statistical Computing; 2017.

[16] Royle NA, Booth T, Valdes Hernandez MC, Penke L, Murray C, Gow AJ, et al. Estimated maximal and current brain volume predict cognitive ability in old age. Neurobiol Aging 2013; 34: 2726-2733.

[17] Reed BR, Dowling M, Farias ST, Sonnen J, Strauss M, Schneider JA, et al. Cognitive Activities During Adulthood Are More Important than Education in Building Reserve. J Int Neuropsychol Soc 2011; 17: 615–24. doi:10.1017/S1355617711000014.

[18] Zahodne LB, Manly JJ, Brickman AM, Siedlecki KL, Decarli C, Stern Y. Quantifying cognitive reserve in older adults by decomposing episodic memory variance: replication and extension. J Int Neuropsychol Soc JINS 2013; 19: 854–62. doi:10.1017/S1355617713000738.

[19] Zahodne LB, Manly JJ, Brickman AM, Narkhede A, Griffith EY, Guzman VA, et al. Is residual memory variance a valid method for quantifying cognitive reserve? A longitudinal application. Neuropsychologia 2015; 77: 260–6. doi:10.1016/j.neuropsychologia.2015.09.009.

[20] Habeck C, Razlighi Q, Gazes Y, Barulli D, Steffener J, Stern Y. Cognitive Reserve and Brain Maintenance: Orthogonal Concepts in Theory and Practice. Cereb Cortex 2017; 27: 3962–9. doi:10.1093/cercor/bhw208.
